# Co-diversification of *Enterococcus faecium* Core Genomes and PBP5: Evidences of *pbp5* Horizontal Transfer

**DOI:** 10.3389/fmicb.2016.01581

**Published:** 2016-10-06

**Authors:** Carla Novais, Ana P. Tedim, Val F. Lanza, Ana R. Freitas, Eduarda Silveira, Ricardo Escada, Adam P. Roberts, Mohammed Al-Haroni, Fernando Baquero, Luísa Peixe, Teresa M. Coque

**Affiliations:** ^1^UCIBIO/REQUIMTE, Laboratório de Microbiologia, Departamento de Ciências Biológicas, Faculdade Farmácia, Universidade do PortoPorto, Portugal; ^2^Servicio de Microbiología, Instituto Ramón y Cajal de Investigación SanitariaMadrid, Spain; ^3^Consorcio de Investigación Biomédica en Red de Epidemiología y Salud PúblicaBarcelona, Spain; ^4^Faculdade de Ciências da Saúde, Universidade Fernando PessoaPorto, Portugal; ^5^Division of Microbial Diseases, UCL Eastman Dental Institute, University College LondonLondon, UK; ^6^Unidad de Resistencia a Antibióticos y Virulencia Bacteriana (RYC-CSIC)Madrid, Spain

**Keywords:** ampicillin resistance, PBP5 mutations, PonA, chromosomal transfer, phylogenomic diversification

## Abstract

Ampicillin resistance has greatly contributed to the recent dramatic increase of a cluster of human adapted *Enterococcus faecium* lineages (ST17, ST18, and ST78) in hospital-based infections. Changes in the chromosomal *pbp5* gene have been associated with different levels of ampicillin susceptibility, leading to protein variants (designated as PBP5 C-types to keep the nomenclature used in previous works) with diverse degrees of reduction in penicillin affinity. Our goal was to use a comparative genomics approach to evaluate the relationship between the diversity of PBP5 among *E. faecium* isolates of different phylogenomic groups as well as to assess the *pbp5* transferability among isolates of disparate clonal lineages. The analyses of 78 selected *E. faecium* strains as well as published *E. faecium* genomes, suggested that the diversity of *pbp5* mirrors the phylogenomic diversification of *E. faecium*. The presence of identical PBP5 C-types as well as similar *pbp5* genetic environments in different *E. faecium* lineages and clones from quite different geographical and environmental origin was also documented and would indicate their horizontal gene transfer among *E. faecium* populations. This was supported by experimental assays showing transfer of large (≈180–280 kb) chromosomal genetic platforms containing *pbp5* alleles, *ponA* (transglycosilase) and other metabolic and adaptive features, from *E. faecium* donor isolates to suitable *E. faecium* recipient strains. Mutation profile analysis of PBP5 from available genomes and strains from this study suggests that the spread of PBP5 C-types might have occurred even in the absence of a significant ampicillin resistance phenotype. In summary, genetic platforms containing *pbp5* sequences were stably maintained in particular *E. faecium* lineages, but were also able to be transferred among *E. faecium* clones of different origins, emphasizing the growing risk of further spread of ampicillin resistance in this nosocomial pathogen.

## Introduction

Infections caused by *Enterococcus* *faecium* have increasingly been reported since the early 1980s’, as have the number of antibiotic resistant isolates of this species (Top et al., 2007). Currently, most clinical strains of *E. faecium* are ampicillin resistant (AmpR), which often also acquire transferable mobile genetic elements, some encoding vancomycin resistance (Arias and Murray, 2012; Wagenvoort et al., 2015). Multidrug resistant *E. faecium* isolates are responsible for infections associated with treatment failures and high mortality rates (Arias et al., 2010; Centers for Disease Control and Prevention, 2013). However, the influence of ampicillin resistance in the population structure of *E. faecium* remains largely unexplored.

*Enterococcus faecium* is intrinsically resistant to cephalos porins and exhibit natural reduced susceptibility to penicillins. This species has six penicillin binding proteins (PBPs) belonging either to class B (monofunctional D,D-transpeptidases), or class A (bifunctional enzymes with glycosyltransferase and D,D-transpeptidase activity), some of them being associated with resistance to β-lactams (Zorzi et al., 1996; Rice et al., 2009). In *E. faecium* resistance to β-lactam antibiotics is conferred by the low-affinity class B PBP5, that requires the participation of class A PBPs (PonA, PbpF) to synthesize the cell wall in the presence of cephalosporins (Williamson et al., 1983; Rice et al., 2009). Resistance to high ampicillin concentrations in *E. faecium* was initially explained by either the enhanced production of PBP5, and/or by polymorphisms in the beta subunit of this protein (Fontana et al., 1996). Soon after, it was demonstrated that such changes were frequently strain specific and did not necessarily correlate with differences in the ampicillin MIC values (Fontana et al., 1996; Zorzi et al., 1996; Rybkine et al., 1998; Rice et al., 2001, 2004; Sifaoui et al., 2001; Belhaj et al., 2016). Further analysis of *E. faecium* strains with different levels of ampicillin susceptibility (0,5- > 128 mg/L) revealed that the variability of PBP5 sequences is mostly due to changes in 21 specific positions of the protein, suggesting that a sequential acquisition of mutations could have contributed to the progressive resistance to ampicillin from the early 1980s (Galloway-Peña et al., 2011; Pietta et al., 2014). Mutations in genes encoding other species-specific proteins that participate in the cell wall synthesis, such as D,D-carboxypeptidases (Ddcp and DdcY), L,D-transpeptidases (Ldt_fm_), glycosyltransferases (PgtA) and acetylmuramoyl-L-alanine amidase (LytG) may also slightly increase the MIC values (Rice et al., 2007; Zhang et al., 2012; Kristich et al., 2014), even in the absence of PBP5 (Sacco et al., 2014). Although the occurrence of β-lactamases has been documented in *E. faecium*, β-lactamases producing *E. faecium* remain rare (Coudron et al., 1992; Klare et al., 1992; Zhang et al., 2012; Hendrickx et al., 2013).

*Enterococcus faecium* population biology is dominated by two main phylogenomic groups, clade A and clade B. Most AmpR isolates belong to clade A or “hospital-associated clade” mainly comprising *E. faecium* from hospitalized patients (Galloway-Peña et al., 2012; Palmer et al., 2012; Lebreton et al., 2013). A subgroup within the clade A, clade A1, is enriched in mobile genetic elements and have enhanced ability to colonize and persist in human hosts due to the presence of adhesins and specific metabolic traits. In contrast, the clade B, or “community-associated *E. faecium*,” mostly comprises ampicillin susceptible (AmpS) isolates from healthy, non-hospitalized individuals (Willems et al., 2005; Lebreton et al., 2013; Tedim et al., 2015; Freitas et al., 2016; Guzman Prieto et al., 2016). The increasing detection of AmpR among isolates from hosts not associated with the hospital setting (Gonçalves et al., 2011; de Regt et al., 2012; Novais et al., 2013; Santos et al., 2013; Tremblay et al., 2013) is of concern, as the acquisition of transferable genes encoding AmpR might facilitate its further spread into AmpS *E. faecium* populations or to other less frequently recovered enterococcal species for which AmpR has been rarely documented (Raze et al., 1998). A relevant observation was the identification of a transferable chromosomal region of 60 kb comprising *pbp5* and a transposon that confers resistance to glycopeptides (*vanB2*-CTn*5386*) (Carias et al., 1998; Rice et al., 2005b). Homologous recombination between similar chromosomal regions carrying *pbp5* of different strains potentially associated with a conjugation mechanism has been recently suggested (García-Solache et al., 2016).

The known diversity of genotypes of AmpR *E. faecium* is mainly focused on the PBP5 polymorphisms analyzed in a limited number of strains (Poeta et al., 2007; Klibi et al., 2008; Galloway-Peña et al., 2011). Similarly, knowledge related to the *pbp5* transferability is limited to the characterization of conjugative Tn*916*-like elements (CTn*5382* or the interaction of CTn*916* and CTn*5386*) in a few *E. faecium* strains (Carias et al., 1998; Rice et al., 2005a,b; García-Solache et al., 2016). Here, we extend previous knowledge on these issues by using a comparative genomics approach of the chromosomal regions containing *pbp5*, that demonstrate a relationship between the diversity of PBP5 and *E. faecium* phylogenomic groups. We also assess the *pbp5* transferability among isolates from different clonal lineages.

## Materials and Methods

### Bacterial Strains

Seventy-eight isolates from a collection of 205 AmpR *E. faecium* (MIC ≥ 16 mg/L) recovered in Portugal from the last decades were analyzed (Novais et al., 2005a,b,c, 2006, 2013). The 78 *E. faecium* were selected in order to include isolates with different antibiotic resistant phenotypes, from diverse origins (39 from different patients admitted in five hospitals from different cities, 18 from swine feces and piggeries, 4 from retail poultry carcasses, 2 from healthy human feces and 15 from hospital wastewater), isolation date and, whenever possible, clonal relatedness. Susceptibility to ampicillin and 10 other antibiotics of different classes was evaluated by disk diffusion and/or agar dilution method (Clinical and Laboratory Standards Institute, 2012). β-lactamase production was tested in AmpR *E. faecium* isolates by the nitrocefin test (5 μl were directly placed in bacteria growing around the ampicillin disk) and PCR amplification of *bla*Z using primers based on the GenBank sequence no. M25257.1 (blaZF-3′-TTGCCTATGCTTCGACTTCA-5′, blaZR- 3′-AGTGAAACCGCCAAGAGTGT-5′). Clonal relatedness was established by Pulsed-Field Gel Electrophoresis (PFGE) and analysis of Multilocus Sequence Typing (MLST) data (Tenover et al., 1995; Homan et al., 2002; Freitas et al., 2013)^1^ using the Bayesian Analysis of Population Structure (BAPS) algorithm (Willems et al., 2012; Tedim et al., 2015).

### Transferability of Ampicillin Resistance

Filter-mating assays were performed in plain Brain Heart Infusion (BHI) not supplemented with antibiotics at 37°C overnight, using a donor/recipient ratio of 1:1 (1-3 experiments per isolate) and *E. faecium* GE1 as the recipient strain. Further filter mating assays under the same experimental conditions but using *E. faecium* BM4105RF and *E. faecium* 64/3 strains as recipients, were performed for those field isolates able to transfer *pbp5* to *E. faecium* GE1. The three laboratory recipient strains used differed in the susceptibility against ampicillin and the presence of *pbp5*: GE1 [Δ*pbp5*; MIC_Amp_ < 0.016mg/L; tetracycline (Tet), rifampicin (Rif) and fusidic acid (Fus) resistant (^R^); ST515/BAPS 2.3b], BM4105RF (*pbp*5; Rif^R^, Fus^R^; MIC_Amp_ = 0.5mg/L; ST172/BAPS 1.3) and 64/3 (*pbp*5; Rif^R^, Fus^R^; MIC_Amp_ = 1mg/L; ST21/BAPS 2.3a). Recipients acquiring *pbp5* are from this point named as transconjugants, based on the most likely transfer mechanism related to *pbp5* (conjugation) published during the revision of this article (García-Solache et al., 2016). Transconjugants were selected on BHI agar supplemented with antibiotics (ampicillin-10 mg/L, fusidic acid-25 mg/L; rifampicin-30 mg/L) and incubated for 24 up to 96 h (37°C) to recover potential transconjugants growing at different times. Transconjugants were confirmed by comparison of their antibiotic resistance phenotype and PFGE profile with those of the donors and the recipients. Stability of acquired *pbp5* chromosomal regions (from now on designated *pbp5* genetic platforms) after serial daily passages (x30) on antibiotic free BHI agar was evaluated in both donor strains and transconjugants. Colonies from each passage were inoculated on plates containing the same agar medium and tested for ampicillin susceptibility by disk diffusion (Clinical and Laboratory Standards Institute, 2012).

### Characterization of the Region Conferring AmpR

Total DNA from a donor strain (AmpR *E. faecium* clinical isolate HPH2), a recipient strain (AmpS *E. faecium* GE1) and the resulting GE1 transconjugant (AmpR *E. faecium* TCGEHPH2.1) was extracted with PureElute^TM^ Bacterial Genomic Kit (EdgeBio, Gaithersburg, MD, USA). DNA concentration was measured with Nanodrop 2000 (Thermo Scientific, Waltham, MA, USA) and Qubit 2.0 fluorometer (Life Technologies). Genomic DNA (250–350 ng) was sequenced on the Illumina MiSeq system using the MiSeq reagent kit v3 and a read length configuration of 2 × 300 bp (Illumina, San Diego, CA, USA). Sequencing was carried out using a standard 2 × 71 base protocol (300–400 bp insert size) in a Genome Analyzer IIx (Illumina, San Diego, CA, USA) at the sequencing facility of the University of Newcastle (United Kingdom). The main statistics for the three sequence datasets (number of reads and coverage) analyzed are shown in Supplementary Table S1. Assembly of sequence data was done using Newbler software (454 life sciences, Roche, Branford, CT, USA). Supplementary Table S2 shows the final assembly results.

Gene prediction was performed using GeneMark.hmm 3.05 (Besemer et al., 2001). Similarity searches for potential protein-coding regions were carried out against a UniRef100 database^2^ using Best Blast Hit approach (Supplementary Table S3). The sequence of the transferred region containing the *pbp5* gene was predicted by a gene-by-gene comparison strategy using *blastn* (genes present in transconjugant strain TCGEHPH2.1 and donor strain HPH2 but not in recipient strain GE1) (Supplementary Table S4). Sequence of the putative “transferred region” was mapped against the genome of the *E. faecium* strain DO (RefSeq Accession: NC_017960) using Easyfig 1.2 software (Sullivan et al., 2011). In addition to *E. faecium* DO, the “transferred region” was also aligned against fully sequenced and closed genomes of *E. faecium* Aus0004, *E. faecium* NRRL B-2354 and *E. faecium* Aus0085 strains (RefSeq accessions numbers: NC_017022, NC_020207 and NC_021994, respectively) with Mauve (Darling et al., 2004). Functional annotation of the query sequences was performed using Blast2GO and Uniprot and KEGG databases^3^

### Analysis of the *pbp5* Genetic Environment

An 8–10 kb DNA fragment comprising the *pbp5* gene and its boundaries was characterized by PCR assays in 15 transconjugants and 21 field isolates (**Table 1**). PCR conditions were adapted according to the expected amplicon size (<3 kb/>3 kb): 0.5/1 mM of each primer, 2/2.5 mM MgCl_2_, 1x of reaction buffer, 0.2/0.4 mM of each deoxynucleoside triphosphate, 1,25U GoTaq^®^ Flexi DNA Polymerase [Promega Corporation, Madison, WI, USA]/2.5U Takara LA Taq polymerase (Takara^TM^ Bio Inc., Shiga, Japan). The amplification program was 25 cycles of 30 s at 94°C, 30 s at 55°C, 30 s at 72°C; 1 cycle 10 min at 72°C (for fragments <3 kb) or 35 cycles of 30 s at 96°C, 1 min at 55°C, 7 min at 72°C; 1 cycle 10 min at 72°C (for fragments >3 kb). Amplicons were further discriminated by comparing the corresponding RFLP patterns obtained after digestion with *Dde*I or *Apa*I and sequences. Genomic location of *pbp5* was identified by hybridization of I-*Ceu*I and *Sma*I-digested genomic DNA using 23S rDNA and/or *pbp5* probes (Liu et al., 1993; Freitas et al., 2013). Labeling and detection were carried out with Gene Images Alkphos direct labeling system kit following the manufacturer’s instructions (Amersham GB/GE Healthcare Life Sciences UK limited). The presence of CTn*916*, CTn*5386* and CTn*5382*, previously associated with transferable AmpR, was determined by analyzing the presence of specific sequences (integrases, excisionases, non-integrase left region) (Carias et al., 1998; León-Sampedro et al., 2016). The transferred 8–10 kb genetic regions carrying *pbp5* characterized in this study were compared with those of all *E. faecium* available genomes at the National Center for Biotechnology Information (NCBI) database (Supplementary Table S5).

**Table 1:** Epidemiological background, *pbp5* gene characterization and antibiotic resistance among *E. faecium* strains and transconjugants from this study.

	*pbp5* genetic environment type (*pbp5*-fstW)				Epidemiological background						
						
Isolate type (isolate name)	(RFLP_*Dde*I)	Sequencing	PBP5 alleles	PFGE*-SmaI pbp5* hybridization (kb)	ST	BAPS	PFGE^a^	Date	Source/sample^d^	MIC to Amp (mg/L)	Resistance to other antibiotics
***E. faecium* recipient strains**							
Rc (*Efc* GE1)	***–***	***–***	***–***	***–***	515	2.3b	A			≤ 0.016	TET, RIF, FA
Rc (*Efm* BM4105RF)	A	I		290	172	1.3	B			0.5	RIF, FA
Rc (*Efc* 64/3)	B	I		250	21	2.3a	C			1	RIF, FA
***E. faecium* donor strains and their corresponding transconjugants**							
WT (H323)	C	I^b^		210	280	3.1	100	2002	H(C)/urine	128	VAN, TEC, ERY, CIP, GEN, STR
TC*Efm*GE1 (GEH323.3)	C	I	C12	210						32	VAN, TEC, TET, ERY, RIF, FA
WT (HPH2)	C	I^b^		210	125	3.1	126	2007	H(A)/urine	128	VAN, TEC, ERY, CIP, NIT
TC*Efm*GE1 (GEHPH2.1)	C	I	C20	210						64	VAN, TEC, TET, ERY, RIF, FA
TC*Efm*64/3 (64HPH2.1)		ND		170 + 250						8	RIF, FA
WT(70411)	D	II^b^		210	670	3.3a	90	1997	H(B)/urine	32	VAN, TEC, TET, ERY, Q/D, STR
TC*Efm*GE1 (GE70411.2)	D	II	C4	210						32	VAN, TEC, TET, ERY, RIF, FA
TC*Efm*BM4105RF (BM70411.5)	D	II	C4	170 + 200						8	VAN, TEC, TET, ERY, RIF, FA
WT (E4)	D	II^b^		210	132	3.3a	Z	2001	HS(E)/water	128	TET, ERY, CIP, GEN, STR
TC*Efm*GE1 (GEE4.1)	D	II	C19	210						16	TET, RIF, FA
WT (28798)	D	III^b^		210	132	3.3a	88	1999	H(B)/blood	64	VAN, TEC, TET, ERY, QD, CIP, GEN, STR
TC*Efm*GE1 (GE28798.1)	D	III	C8	210						32	VAN, TEC, TET, ERY, STR, RIF, FA
WT (E233)	D	III^b^		210	132	3.3a	88.1	2002	HS(E)/water	64	ERY, CIP, GEN, STR
TC*Efm*GE1 (GEE233.1)	D	III		210						32	TET, RIF, FA
WT (E169)	D	III^b^		210	132	3.3a	88.2	2001	HS(C)/water	64	ERY, CIP, GEN, STR
TC*Efm*GE1 (GEE169.3)	D	III		210						32	TET, RIF, FA
TC*Efm*BM4105RF (BME169.3)		ND		200 + 240						8	RIF, FA
WT (E49)	D	III^b^		210	132	3.3a	C3	2001	HS(E)/water	128	VAN, TEC, ERY, CIP, STR
TC*Efm*GE1 (GEE49.1)	D	III	C8	210						32	VAN, TEC, TET, RIF, FA
WT (H207)	ND	ND	C4	210	280	3.1	150	2002	H(C)/exsudate	32	TET, ERY, CIP, GEN, STR
TC*Efm*GE1 (GEH207.1)	ND	Unknown^c^		180						32	TET, RIF, FA
TC*Efm*BM4105RF (BMH207.3)	ND	ND		210						8	RIF, FA
WT (SN71, SN133)	C	I^b^		200	393	2.1a	SN208	2006	PG/n = 4-waste lagoon, animal faeces	64	TET, ERY, STR, NIT
TC*Efm*GE1 (GESN71.1; GESN133.1)	C	I	C9	200						64	TET, RIF, FA
TC*Efm*BM4105RF (BMSN71.1)	C	I		200						8	RIF, FA
TC*Efm*64/3 (64SN71.1)	C	I		210						64	RIF, FA
WT (VD79C1)	D	II^b^		200	18	3.3a	59	2001	HH/faeces	128	VAN, TEC, TET, ERY, CIP, GEN
TC*Efm*GE1 (GEVD79C1.5)	D	II	C7	210						128	VAN, TEC, TET, ERY, GEN, RIF, FA
TC*Efm*BM4105RF (BMVD79C1.6)	D	II	C7	200						128	VAN, TEC, RIF, FA
**Representative *E. faecium* without *pbp*5 transfer**							
WT (529940)	ND	II	C6	ND	16	3.3a	74	2000	H(B)/urine	> 64	VAN, TEC, TET, ERY, STR
WT (H352)	ND	II	C4	ND	280	3.1	71	2000	H(D)/sputum	> 32	VAN, TEC, ERY, CIP, GEN
WT (HPH6)	ND	II	C5	ND	18	3.3a	128	2007	H(A)/pus	> 32	VAN, TEC, ERY, CIP, NIT
WT (E197)	ND	III	C8	ND	368	3.3a	H4	2001	HS(C)/water	> 32	VAN, ERY, CIP, GEN, STR
WT (SN446)	ND	III	C8	ND	132	3.3a	H119.5	2007	PG/ water	> 32	VAN, TEC, ERY, GEN
WT (H196)	ND	V	C1	ND	390	3.1	129	2002	H(D)/unknown	> 32	TET, ERY, STR
WT (C373)	ND	I	C11	ND	148	2.1b	36.2	2001	HH/faeces	> 32	TET, ERY, CIP, GEN, STR, NIT
WT (SE97F1)	ND	I	C10	ND	*pur*K6	ND	ND	2001	P/skin	> 32	TET, ERY, CIP, STR
WT (164306)	ND	V	C2	ND	190	2.3a	98	1998	H(B)/urine	32	VAN, TEC, TET, ERY, CIP, STR
WT (SN194)	ND	V	C3	ND	264	2.3a	ND	2006	PG/residual water	32	TET, CIP, CHL, STR


*^a^Each different PFGE number or letter represent a different clone. In clones 88.1 and 88.2, the number after a dot represents the number of bands differing from the more ancient clone 88. ^b^The *pbp5* genetic platform type of these wild strains was determined by RFLP, which pattern was the same as the respective transconjugants with *pbp5* platform characterized by sequencing. ^c^In this strain we could not amplify the region between *pbp5* and *fstW* or *pbp5* and *N*-acylglucosamine-6-phosphate 2-epimerase. Abbreviations: Rc, recipient strain; WT, wild type; TC, transconjugant; Efm, *E. faecium*; H, hospital; HS, hospital sewage; HH, healthy human; PG, piggery; P, poultry; VAN, vancomycin; TEC, teicoplanin; TET, tetracycline, ERY, erythromycin; Q/D, quinupristin-dalfopristin; CIP, ciprofloxacin; STR, High level resistance (HLR) to streptomycin; GEN, HLR to gentamicin; NIT, nitrofurantoin; RIF, rifampicin; FA, Fusid Acid; ND, not determined. ^d^Letters appearing in parenthesis of H (hospitals) and HS (hospital sewage) represent the five hospitals included (A-E).*

### Phylogenetic Analysis of PBP5

Comparative analysis of all PBP5 protein sequences available in the GenBank database with those identified in this study was performed using the basic local alignment sequence tool (BLAST) (Altschul et al., 1997) and the ClustalW2 program for multiple sequence alignment (Larkin et al., 2007; Goujon et al., 2010), hosted by the National Center for Biotechnology Information (NCBI) and the European Bioinformatic Centre (EBI), respectively. The PBP5 amino acid sequences were designated by a “C” followed by a number in agreement with the nomenclature used in previous works (Supplementary Table S6) (Galloway-Peña et al., 2011). Multiple sequence alignments of all PBP5 sequences were performed through MEGA 7 software^4^ and the topology of the phylogenetic tree was inferred by maximum-likelihood algorithm using PhyML (Guindon et al., 2010) with bootstrap analysis based in 1000 permutations and a cut-off of ≥70%.

Phylogenetic congruence of core genomes and *pbp5* sequences was established by comparative analysis of their gene tree topologies. Core genomes for the 233 *E. faecium* strains whose draft genome sequences were available at NCBI Trace Database (last updated on February 2014), were reconstructed independently using an all-against-all reciprocal BLAST approach. Briefly, each CDS was used as a BLAST query against a local database of CDS from all genomes in the sample analyzed. Afterward, we re-annotated the chromosome sequences with GeneMarkS v2.8 (Besemer et al., 2001). The core genome was defined as the non-redundant genes present in all strains using CD-HIT (Li and Godzik, 2006) with the 80% coverage, 80% identity cut-offs, and parsed by homemade Perl script. All core genome genes were concatenated and aligned using MAFFT (Katoh and Toh, 2010). The phylogenetic tree was built by FastTree2 (Price et al., 2010). Tree comparison was carried out by *cophyloplot* command of APE (Analyses of Phylogenetics and Evolution) (Paradis et al., 2004) using R software.

## Results

### Transferability of AmpR (*pbp5*)

Twelve isolates (*n* = 12/78; 15%; **Table 1**) were able to transfer under our experimental conditions a chromosomal genetic platform containing *pbp5* (associated with AmpR) to *E. faecium* strain GE1. Five of them were also able to transfer *pbp5* to *E. faecium* BM4105RF and two, to *E. faecium* 64/3. All donor strains belonged to major *E. faecium* human lineages mostly associated with clinical isolates causing hospital infections, namely BAPS subgroups 3.3a (ST670, ST132, ST280), 3.1 (ST280, ST125), and 2.1a (ST393). They were obtained from samples of hospitalized humans but also from samples of hospital sewage, healthy humans and piggeries. β-lactamase production was not detected in any of the isolates.

**FIGURE 1:**
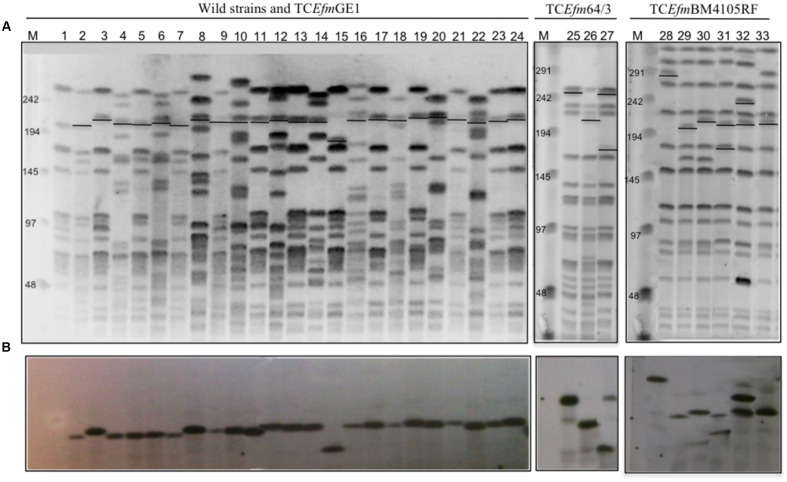
**Clonality and hybridization assays with *pbp5* probes of wild type, recipients and transconjugants strains.** **(A)** PFGE SmaI digested DNA of recipient strains (1-*E. faecium* GE1, 25-*E. faecium* 64/3, 28-*E. faecium* BM4105RF), wild type (2-VD79C1, 4-SN71, 6-SN133, 8-HPH2, 10-H323, 12-70411, 14-H207, 16-E49, 18-E233, 20-E169, 22-E4) and transconjugants (3-GEVD79C1.5, 5-GESN71.1, 7-GESN133.1, 9-GEHPH2.1, 11-GEH323.3, 13-GE70411.2, 15-GE207.1, 17-GEE49.1, 19-GEE233.1, 21-GEE169.3, 23-GE28798.1, 24-GEE4.1, 26- 64SN71.1, 27- 64HPH2.1, 29- BMSN71.1, 30- BMH207.3, 31- BM70411.5, 32- BME169.3, 33- BMVD79C1.6). **(B)** Hybridization assays with a *pbp*5 probe (primers P1 and P2-**Figure 4**). M- Low Range PFGE Marker, kb (New England, BioLabs). Abbreviations: TC*Efm*- Transconjugant *E. faecium.*

All transconjugants had similar PFGE profiles to recipient strains (**Figure 1**), were resistant to rifampicin and fusidic acid and exhibited a variable susceptibility to ampicillin (MICs = 8–128 mg/L), which was often lower than that of their corresponding donor isolates (8 mg/L or 16 vs. 32 mg/L to >256 mg/L). Besides the AmpR phenotype, some transconjugants also exhibited resistance to vancomycin (*n* = 8), teicoplanin (*n* = 8), erythromycin (*n* = 6), tetracycline (*n* = 1; in non-GE1 transconjugants; as GE1 strain is tetracycline-resistant), streptomycin (*n* = 1) and/or gentamicin (*n* = 1). Transconjugants carrying the *pbp5* genetic platform either alone or with plasmids harboring genes encoding resistance to different antibiotics were recovered from selection plates supplemented with ampicillin and not with other antibiotics (data not shown).

### The *pbp5* Gene of AmpR Isolates Is Located on Large and Transferable Chromosomal Genetic Platforms Containing Metabolic Traits

Hybridization of *pbp5* and 23S rDNA probes with a digested I-*Ceu*I DNA band of high MW revealed a chromosomal location of *pbp5*. Further hybridization of *Sma*I-digested genomic DNA with the *pbp5* probe showed hybridization to fragments of ∼210 kb in all but one GE1 transconjugants, for which the *pbp5* probe hybridized to a band of ∼180 kb (**Figure 1**). Hybridization of the same probe with fragments of *Sma*I-digested genomic DNA of *E. faecium* BM4105RF and *E. faecium* 64/3 transconjugants of different sizes reflects independent transfer events. Similarity in the PFGE patterns of the transconjugants obtained using different donors indicates that DNA acquisition occurs within a particular region of the genome (**Figure 1**). The donor strains lack integrases/excisionases of Tn*916*-like conjugative transposons (Tn*916*, Tn*5386*, Tn*5382*).

**FIGURE 2:**
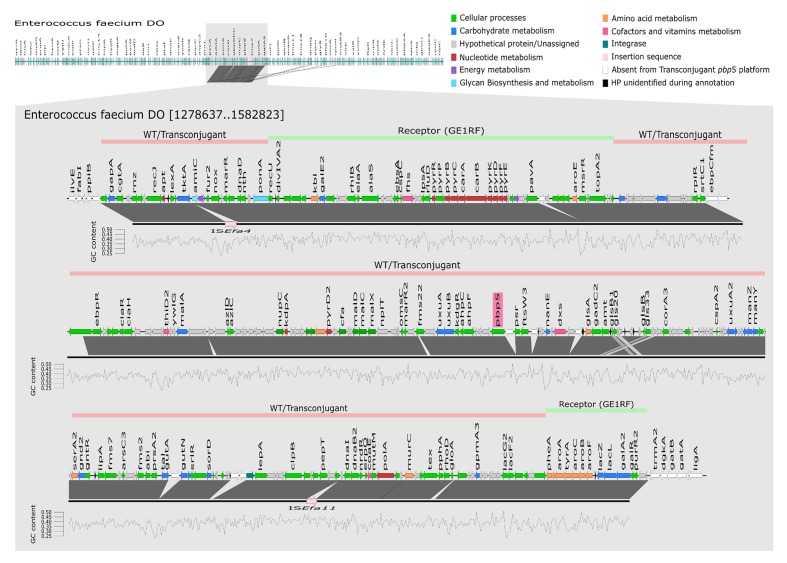
**Representation of the transferable chromosomal genetic platform containing *pbp5*.** Mapping and annotation (using KEGG database) of transferable *pbp5* genetic platform of transconjugant TCGEHPH2.1 (represented by black line) using *E. faecium* DO as reference genome. Lines above *E. faecium* DO genetic structure represent the transferable *pbp5* genetic platform observed in wild type and transconjugant isolates (pink) and genomic regions of *E. faecium* GE1 recipient and transconjugant (green). GC content was calculated using seqinr in Rstudio. The window used to calculate the GC content was 200 bp, represented in the figure by each vertices of the graph. WT, wild type.

**FIGURE 3:**
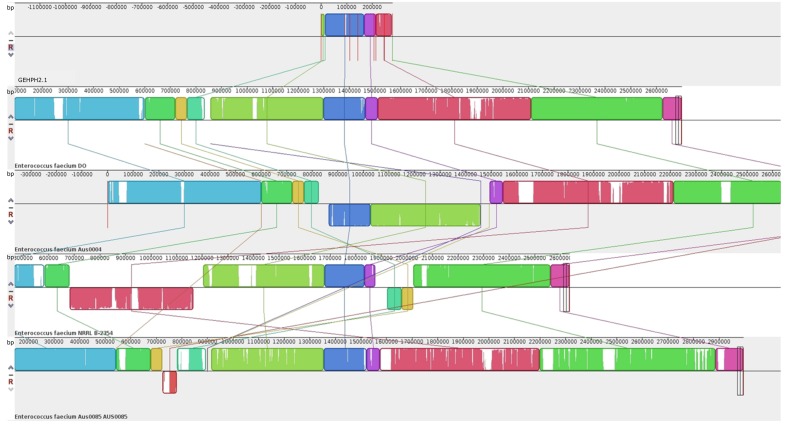
**Comparison by MAUVE of the transferable genetic platform containing *pbp5* (TCGEHPH2.1) with the four close *E. faecium* genomes (DO, Aus 0004, Aus0085 and NRRL B-2354), present in GenBank database.** Each color block represents a genome region present in at least two of the sequences analyzed. These similar blocks can be identified by their color. Blank regions within the block represent point mutations or even regions that are absent in the same color blocks of the other sequences.

For whole genome sequence a representative donor:recipient pair and its transconjugant were analyzed. The donor was the clinical HPH2 strain; the recipient, *E. faecium* GE1 which did not originally carry *pbp5*, and thus limiting the possibilities of recombination events between the acquired and natural *pbp5* genetic platform in the recipient strain; and the size of the *Sma*I digested genomic DNA fragment, the one that most commonly hybridizing with the *pbp5* probe (∼210 kb). Sequencing of the recipient (*E. faecium* GE1), the donor (*E. faecium* HPH2) and the transconjugant (*E. faecium* TCGEHPH2.1) allowed us to identify 7 contigs with genes present in the donor and its transconjugant but absent in the recipient strain. The fragments which comprised the *pbp5* gene, represent a genetic platform of approximately 280 kb that is slightly larger than that inferred from the *Sma*I-PFGE gels. A more detailed analysis of the contigs revealed that two of them (contig00158 and to a lesser extent contig00068) also contained genes present in the recipient and transconjugants but not in the donor strain, which could be explained either by the partial transfer of the *pbp5* genetic platform and/or post-transfer recombination events (**Figure 2**). The comparative analysis of the genomes sequenced in this work with the four closed *E. faecium* genomes available at the NCBI database at the time of writing this manuscript [DO (NC_017960.1), NRRL B-2354 (CP004063.1), Aus0004 (NC_017022.1), Aus0085 (NC_021994.1) strains], revealed a common region of 153kb for our donor/transconjugant strains and the four genomes analyzed, which was absent in the recipient *E. faecium* GE1 genome (**Figure 3**). The remaining sequence of the 280 kb “transferred chromosomal region” was variably present and located in different genomes (**Figure 3**).

**Figure 2** shows a detailed characterization of the 280 kb transferred genetic platform, with ORFs of a G + C content ranging from 24 to 41%. Besides the *pbp5*-related resistance to β-lactams, this chromosomal transferable genetic platform contains genes involved in different cellular functions including amino acids and carbohydrate transport, redox processes, survival under stressful conditions in the intestinal environment (e.g., acid and bile tolerance) and also other genes related to β-lactam resistance. Among the last category is a gene (named *ponA*) encoding a bifunctional class A PBP with transpeptidase and transglycosylase activity, which is involved in the synthesis of peptidoglycan, allowing the survival in the presence of cell wall inhibitors (Rice et al., 2009). A copy of the CiaRH operon, coding for a two-component signal-transduction system responsive to cell envelope lysis-stress response and restricted to *Streptococcus* spp. to date, was also detected. The 280 kb genetic platform containing *pbp5* also had genes with presumed influence on microbe-host interactions. These include three phosphotransferase systems (PTS, namely glucitol/sorbitol, L-ascorbate and mannose/fructose/sorbose), the ABC transport and transformation system of maltodextrines (*malACDX*) and the *N*-acetylmannosamine-6-phosphate epimerase (NanE; part of a pathway that allows the usage of sialic acids, major components of glycoproteins, gangliosides, and other sialoglyco-conjugates). Other metabolic genes in the *pbp5* genetic platform could be involved in tolerance to the intestinal acidic environment, including glyceraldehyde-3-phosphate dehydrogenase, ATP synthase subunit α, NADH dehydrogenase, glutaminase and genes encoding enzymes involved in the production of ammonia from glutamine and deamination or transport of branched/nitrogenated amino acids. Finally, five stress response proteins including two belonging to the Csp (cold shock proteins) system (CspA and CspC) and the small chaperone Hsp20, and Gls33 (also present in *E. faecium* genomes available in the GenBank databases) were also identified. They are involved in stress responses to salts, pH and ethanol exposure in *Clostridium*. Hsp20 is a small chaperone protein involved in survival of different abiotic stress conditions including heat (55°C) and salt (5 mM) in *Bifidobacterium longum* (Khaskheli et al., 2015).

To date, only two *E. faecium* strains lack *pbp5*. These are strains GE1 and D344SRF, which are AmpS isolates. The D344SRF strain is susceptible to cephalosporin and ampicillin due to the spontaneous deletion of a 170 kb genome fragment that includes *pbp5* and other *pbp* genes. This deletion occurred by the interaction of CTn*5386* (a 60 kb element that comprises *pbp5* and *vanB2*) with Tn*916* (Rice et al., 2007). The GE1 laboratory strain does only harbor the integrase of Tn*5801.* The causes of the loss of the *pbp5* genetic platform are still unknown.

### The Chromosomal Region Harboring *pbp5* Exhibit Hotspots for Insertions

**FIGURE 4:**
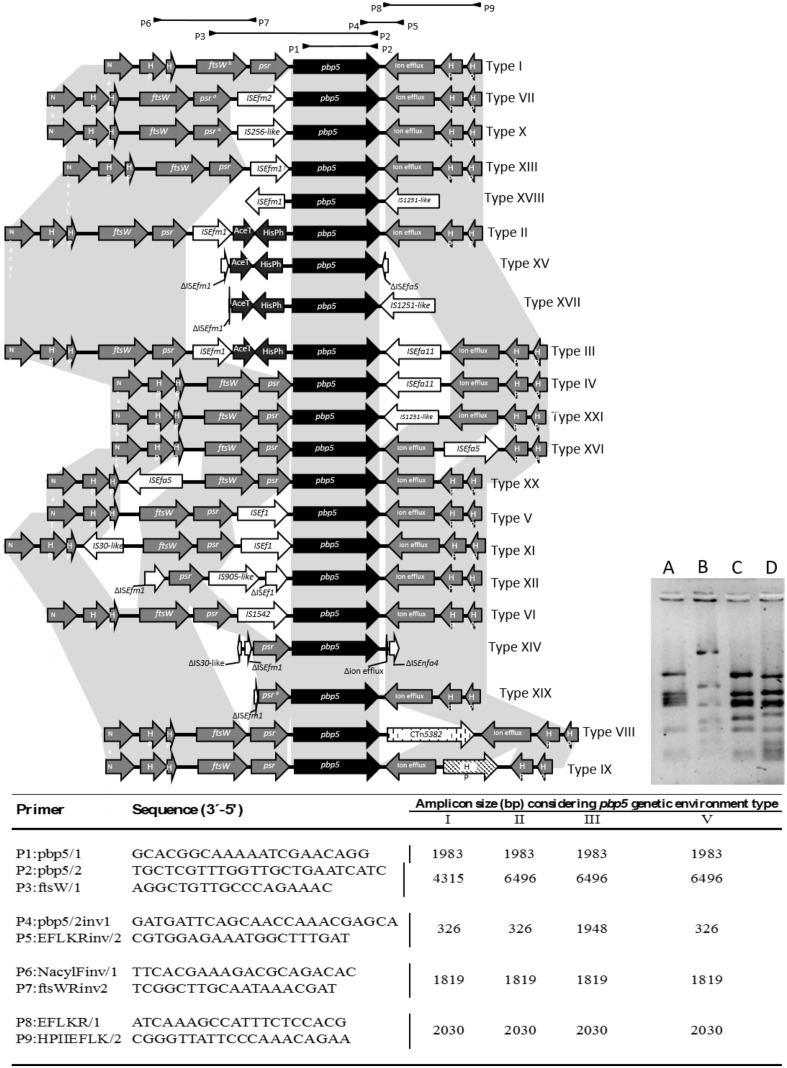
**Characterization of *E. faecium pbp*5 genetic environment by PCR and sequencing.** The Roman numbers I, II, III, and V represent the different *pbp5* genetic platforms detected in *E. faecium* from this study. The numbers IV, VI to XXI were detected in available genomes from GenBank database. The different types were named according to diversity of insertions sequences, genomic fragments or conjugative transposons within genes or intergenic regions. Mutations or recombinations within genes or intergenic regions were not considered for type classification. The Table indicates the primers used (designed for this study; P1/P2 described by Dahl et al., 2000) and the size of PCR amplicons from genetic environment of types I–III and V. The A, B, C and D lines of the bottom right side figure represent RFLP patterns of amplicon P3-P2 of mobile platforms I (pattern C) and II/III (pattern D) of isolates included in this study, when digested with DdeI restriction enzyme. The patterns A and B correspond to the amplicons of the recipient strains *E. faecium* BM4105RF and 64/3, respectively. ^a^ These gene has an extra stop codon within its sequence. Abbreviations: *N*-acyl, (*N*-acyl-glucosamine-6-phosphate-2-epimerase); HP (hypothetical protein); *ftsW* (cell cycle protein); *psr*, (*pbp5* synthesis repressor); *pbp5* (gene encoding penicillin binding protein 5); AceT (acetiltransferase); HisPh (Histidinol Phosphate Phosphatase).

The occurrence of different insertion sequences (ISs), (**Figure 4**) in the boundaries of the *pbp5* gene and the presence of a >3027 bp *psr-pbp5* fragment in *E. hirae* (99.9% identical to that found on *E. faecium*) suggest the presence of hot spots that could facilitate recombination of regions containing the *pbp5*. To test this possibility, we further analyzed the 8–10 Kb genetic environment of *pbp5* from 21 wild type and 15 transconjugant isolates included in this study as well as available enterococcal genomes. Identical RFLP patterns of these 8–10 kb fragment were observed for each pair of donor and its corresponding transconjugants, but were different from those naturally occurring in the *pbp5* carrying recipient strains *E. faecium* BM4105RF and 64/3 (**Figure 4**). Sequencing of fragments representing distinct RFLP patterns and comparative analysis with similar *pbp5* genetic environment in GenBank available genomes, revealed 21 variants of such 8–10 kb chromosomal fragments (designated by roman numerals), which differed in the number and type of ISs (**Figure 4**). Three variants (types I, II, III) corresponded to *pbp5* transferable platforms described in this study (**Figure 4**; **Table 1**).

**FIGURE 5:**
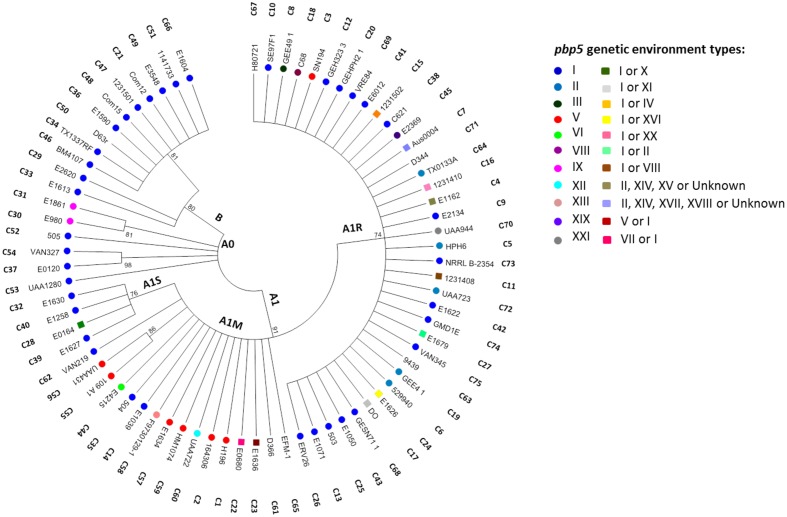
**Phylogenetic analysis of PBP5 protein sequences from *E. faecium* isolates of this study and available in GenBank database (last update February 2014).** The Maximum Likelihood tree was obtained using Mega 7 and the JTT method. The tree with the highest log likelihood (-2000,3736) is shown. Initial tree(s) for the heuristic search were obtained automatically by applying Neighbor-Join and BioNJ algorithms to a matrix of pairwise distances estimated using a JTT model. Then, the topology with superior log likelihood value was selected. The analysis involved 75 amino acid sequences. All positions containing gaps and missing data were eliminated of the analysis. The final dataset had a total of 445 positions. Evolutionary analyses were conducted in MEGA7. Bootstrap values are indicated and are based in 1000 permutations. The cut-off used was ≥70%. “C” followed by a number represent PBP5 amino acid combinations described in Supplementary Table S6. Only one *E. faecium* isolate carrying representative PBP5 sequences (each type of “C”) was included in the tree. The *pbp5* genetic environments types described in **Figure 4** are indicated by colored circles or by colored squares when more than one type was detected in isolates carrying the same “C.” No symbol reflects that the genetic environment was not elucidated. NA, not applicable.

The predominant 8–10 kb fragment identified in both AmpR and AmpS *E. faecium* analyzed in this study did not contain indels and is considered here as the *pbp5* genetic environment prototype; arbitrarily named Type I (Supplementary Table S5, **Figure 4** and **5**). The *pbp5* or other genes (e.g., *psr. ftsw*, ion efflux genes) were flanked by one or two insertions sequences of the IS*256*, IS*L3* or IS*982* families (IS*Ef1*, IS*Efm1*, IS*Efm2*, IS*1542*, IS*256*-like, IS*Efa11*, or IS*1251*-like) in isolates carrying types II to XXI, with the exception of type VIII that had CTn*5382* (*vanB2*) inserted downstream of *pbp5*. The identifiable boundaries of ISs detected within the 8–10 kb boundaries of *pbp5*, as well as the common nucleotide positions at which insertions occurred, suggested recent acquisition events at some hot-spots on an ancestral sequence (**Figure 4**). Despite of predominance of type I, some types seem to be more associated with specific hosts as the case of type V, predominant in strains from pigs and type II/II-like, III or type XIV-like which are frequent among clinical isolates. Epidemiological distribution of isolates appears in Supplementary Table S5.

### Diversity of PBP5 Sequences Reflects the Phylogenomic Diversification of *E. faecium*

We identified 75 PBP5 protein variants (Supplementary Tables S5 and S6) corresponding both to AmpR and AmpS strains which comprise 20 of the previously described variants C4, C7, C9, C11, C15-C18, C21, C24, C46-C48, C50, C51, C61, C63, C65, C67, C71 (Rybkine et al., 1998; López et al., 2009; Galloway-Peña et al., 2011). The other 55 variants were firstly detected in this study either in strains from Portugal (*n* = 11) or from available genomes at NCBI database (*n* = 44). The C4, C7, C8, C9, C12, C19, and C20 sequences were identified in isolates able to transfer *pbp5* (**Table 1**, Supplementary Table S6; **Figure 5**), which belonged mostly to ST18, its SLV ST125, ST280 and ST670 (BAPS 3.3a, 3.1 and 2.1a, respectively) (**Table 1**).

**Figure 5** shows the phylogenetic tree constructed with all PBP5 protein sequences from this study and those available at GenBank databases. The tree is split into two major clades arbitrarily named B and A, mirroring the clades associated with populations of non-hospitalized persons and hospital isolates respectively, which were previously inferred from phylogenomic studies of *E. faecium* (Lebreton et al., 2013). Clade B comprises PBP5 of AmpS isolates (PBP5-S), which mainly correspond to strains of BAPS subgroups 1.2 and 1.5. Some are similar to the prototype PBP5-S C46 sequence of *E. faecium* BM4107 strain (Sifaoui et al., 2001) but most of them also exhibit mutations at positions T25A, S39T and D644N, which are also common to PBP5 sequences of clade A isolates (Supplementary Table S6). Two strains, isolated in 1964 and 2006, further showed changes at S27G + T324A and S27G respectively, such mutations corresponding to the PBP5-R consensus sequence (Pietta et al., 2014). The type I chromosomal region mentioned above was observed in all available isolates from this group (**Figure 5**; Supplementary Table S5).

Clade A includes PBP5 variants grouped in two main clusters arbitrarily designed here A0 and A1, with most isolates sharing mutations at positions V24A, S27G, E100Q, K144Q, T172A, T324A, N496K, A499T and E525D (7 of the 21 positions used to establish sequence diversity of this protein) (Pietta et al., 2014). The Clade A0, represented by C30, C31, C37, C52, C53, and C54 PBP5 sequences, corresponds to AmpS isolates of different BAPS groups (BAPS 1.2, BAPS 2.3a and BAPS 3.3b), recovered from different hosts (animals and humans), different countries and collected from 1995 to 2001 (**Figure 5**, Supplementary Table S6). Most isolates within clade A0 carried *pbp5* within a type I fragment. Sequences of Clade A1 were classified in three subclusters that parallels *E. faecium* populations of BAPS groups 2 and 3 (Willems et al., 2012; Tedim et al., 2015). They were designated as A1S, comprising a subset of PBP5-AmpS variants (mainly associated with human and animal isolates of BAPS subgroup 2.1b); A1M, comprising a subset of PBP5-AmpS and PBP5-AmpR (mostly associated with animal isolates of diverse BAPS subgroups 2.1b, 2.3a, 2.3b, 3.1, 3.2, and 3.3b); and A1R, including almost all PBP5-AmpR (mostly associated with the clinical setting and BAPS subgroups 3.1, 3.3a and 2.1a, the latter two only observed in this group).

All isolates from Clades A1S, A1M and A1R share mutations R34Q, G66E, L177I and A216S but also present some differences. Mutations S39N and A401S plus A499I were specific for subclades A1S and A1M, respectively. The last two polymorphisms were previously documented in two AmpR strains (Pietta et al., 2014) but this study suggests that they were fixed in certain populations despite having been previously discarded as relevant changes. One of the A1M strains had also variations at specific positions linked to the A1R group (A68T, E85D, M485T, V586L, E629V and P667S). Of note is the PBP5 variant C23 within this cluster, which was overrepresented (*n* = 44).

PBP5 variants within the A1R group exhibited six mutations (A68T, E85D, S204G, 466′S/D, M485A/T, E629V, P667S) predominant in this group, some of them located in the active site of the protein (466′S/466′D, M485A/T) and in the end of a turn between the β1 and β2 strands (E629V; P667S) (Fontana et al., 1996; Rice et al., 2004). It is of note that some AmpS isolates (BAPS subgroups 3.1, 2.3a and 3.2) with PBP5 sequences that cluster in the A1R subgroup (including the PBP5 of the recipient *E. faecium* 64/3) lack the mutation M485A and E629V, which confirmed that such mutations might be necessary for AmpR phenotype, as reported (Rice et al., 2004). Changes A68T, E85D and S204G were shared by AmpS and AmpR isolates of A1R group (Supplementary Table S6). Some strains exhibited the particular pattern of mutations Q408H, A558T, G582S, K632Q and, eventually, V462A, N546T and P642L. They had not previously been associated with AmpR. Distribution of different variants of the 8–10 kb boundaries of *pbp5* gene is shown in **Figure 5** and Supplementary Table S5. Of note, is the detection of type I in isolates of different groups of clade A1 as well as the association of types V and XI with isolates of group A1M and of the similar types II, III, XIV, XV, and XVII with isolates of group A1R.

We also analyzed the diversity of other genes previously associated with AmpR (*ddc*P, *ddc*Y, *ldt*Efm, *pgt*A, *lytG*) in available genomes, (protein sequence per UniRef100 available at Uniprot using as reference *E. faecium* strain Aus0004). Single-locus phylogenetic tree of PBP5 was congruent with those of proteins codified by *ldt*Efm and *ddcY* but was non-congruent with those of *ddcP, pgtA* and *lytG* (data not shown).

**FIGURE 6:**
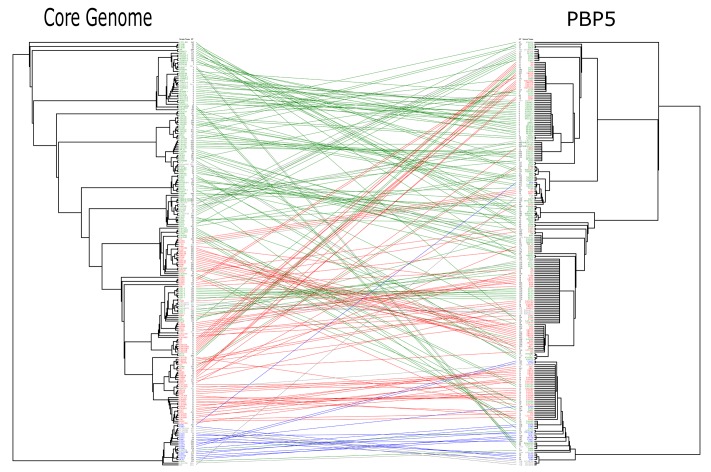
**Comparison of gene tree topologies (Core Genome phylogeny vs. *pbp5* phylogeny).** Strains of clade B are represented in blue, those of Clade A1 in red and those Clade A2 in green. The edges join the core genome and the corresponding *pbp5* of each strain. Both trees were made by ML using GTR-CAT. Tress and edges were built using APE packages. The results of Mantel test (*r* = -0.03 and significance of 0.761) show the rearrangement of the *pbp5* in comparison with the core genome. The Mantel test (APE package) was made using the distance matrix calculated from nucleotide alignments.

Analysis of gene tree topologies (*E. faecium* core genomes vs. *E. faecium pbp5* genes) revealed discrepancies that indicate transfer of *pbp5* between *E. faecium* populations (**Figure 6**) and explains the occurrence of both the same PBP5 variants in isolates of different clonal lineages, and different PBP5 sequences in strains of the same phylogenomic groups (**Figure 5**, Supplementary Table S5).

### Ampicillin Resistance Was Maintained Without Selective Pressure

AmpR was maintained in the wild-type strains and respective *E. faecium* GE1 transconjugants over 200 generations in antibiotic free BHI indicating that the acquired genetic platforms containing *pbp5* may persist in absence of selective pressure in different genetic backgrounds, suggesting no-fitness cost imposed by the acquired *pbp5* platforms. The morphology and growth rate of all evolved transconjugants was similar among each other and to the normal growth of the *E. faecium* GE1 recipient strain.

## Discussion

This study documents the occurrence and diversity of a large chromosomal region containing *pbp5* in almost all *E. faecium* genomes and a parallel diversification between the PBP5 and the *E. faecium* core genome. The transferability of this region observed under laboratory conditions using different clonal backgrounds would greatly enhance the possibilities of *E. faecium* adaptation to changing environments.

Polymorphisms in the PBP5 protein sequences allowed grouping of PBP5 variants in clusters that mimic the phylogenomic diversification of *E. faecium* (Galloway-Peña et al., 2012; Lebreton et al., 2013). Lebreton et al. (2013) suggested a model for evolvability of this enterococcal species consisting of a first split of “clade B” and “clade A” coincidental with human and animal co-habitation occurring 30,000 y ago, and a further split of “clade A” in subclades “A1” and “A2” after the introduction of antibiotics in the late 1940s. Two main clusters of PBP5 variants were also identified in the present study, designated as “B” (associated with the *E. faecium* “clade B”), and “A” that further split in subgroups A0 (including only AmpS isolates) and A1 comprising three small groups linked to different *E. faecium* populations differing in the susceptibility to ampicillin: A1S (AmpS from healthy humans of different BAPS groups), A1M (AmpS and AmpR from humans and animals), and A1R (AmpR from clinical isolates). Based on the apparent universal presence of *pbp5* in *E. faecium* populations, we could speculate that *pbp5* might have predated the *E. faecium* evolutionary split among different hosts. Although some authors have suggested a sequential acquisition of amino acid changes in PBP5 sequence (Galloway-Peña et al., 2011; Pietta et al., 2014), such diversification may indicate different evolutionary routes for AmpR in response to distinct selective pressures in different hosts, similarly to what has been observed for different β-lactamase enzymes of Gram negative organisms (Novais et al., 2010).

The full characterization of the transferable 153 kb chromosomal region that contains not only *pbp5* but also genes contributing to survival in the gastrointestinal tract (e.g., resistance to acids and bile) in almost all available *E. faecium* genomes suggest a contribution of this genetic platform to the adaptation to the mammalian intestine and persistence in abiotic environments. Due to the scarcity of *E. faecium* isolates from non-mammals and non-human hosts, it would result highly speculative to hypothesize about the origin of the PBP5 and other genes located in this genetic platform. The PBP5 is a member of the Mec family, which comprises proteins with natural low affinity for β-lactams as PBP2a (*mecA*) of *Staphylococcus aureus* and PBP3r of *Enterococcus hirae* (99% identical to PBP5, suggesting a common origin for PBP5 and PBP3r) (Raze et al., 1998; Hiramatsu et al., 2013). For staphylococci, MecA would have been essential for the survival of ancestral members of staphylococci in the presence of β-lactam antibiotics produced by fungi and Actinobacteria and lost in the Devonian period with the emergence of mammals (more than 400 million of years ago). This would be coincidental with the separation and adaptation of staphylococcal species to different hosts (Hiramatsu et al., 2013), in any case isolating *Staphylococcus* populations from old β-lactam producers. Recent antibiotic pressure would have influenced the transference of PBP2a from environmental to human strains of staphylococci. It is of note that the 153 kb transferred genetic platform characterized in this study has a variable G + C content, reflecting HGT events from different sources and suggesting that a similar evolutionary route as that of PBP2a of staphylococci might have also occurred for *E. faecium* PBP5.

The transfer of *pbp5* in *E. faecium* resulting of the interaction between transposons as Tn*5382* or Tn*916* plus Tn*5386* was previously demonstrated for a few isolates (Rice et al., 2005a). During the revision process of this manuscript, a work by García-Solache et al. (2016) was published. This work suggested that transference of chromosomal regions carrying *pbp5* occurs by homologous recombination between similar DNA regions of donor and recipient strains enriched in AT nucleotides with a possible involvement of plasmids (García-Solache et al., 2016). Tn*916*-like elements were not detected in our strains but the presence of a band of similar size in both donors and recipients and a sequence enriched in AT in the boundaries of the chromosomal transferred region (data not shown) suggested that homologous recombination might occur between *E. faecium* donor and recipient DNA from our study. Although the presence of plasmid genes within this acquired *pbp5* genetic platform was not observed in the transconjugant we sequenced as well as in the case of some transconjugants described in the study of García-Solache et al. (2016), we cannot completely discard this possibility due to the common presence of plasmids of certain families in donors analyzed (data not shown) (Manson et al., 2010; Freitas et al., 2016).

Despite the relevance of elucidation of the mechanism of *pbp5* transfer at molecular level in future studies, it is of note that disparate topologies of the phylogenetic trees of *E. faecium* core genomes and PBP5 sequences obtained in our study reflect how frequent the transfer of *pbp5* genetic platforms under natural circumstances may occur (Tripathi and Sowdhamini, 2008). Similarly, atypical phylogenetic associations have been signaled as evidence of lateral transfer of various types of serine-proteases (PBP5 belongs to this family of enzymes) throughout the prokaryotic world (Tripathi and Sowdhamini, 2008). In fact, we present evidence of inter-chromosomal transfer for different PBP5 C-types. However, in our case, *pbp5* horizontal gene transfer (HGT) events seems to preferentially happen in particular populations, which being more prone to mutation (the subclade A1 that is characterized by a higher mutation rate than clade B) would further facilitate the *pbp5* fixation and evolution followed by their clonal expansion under antibiotic selective pressure, reflecting the “*ex unibus plurum*” evolutionary dynamics (Baquero, 2011). Heterogeneity of populations that colonize humans, designated as “clouds” elsewhere (Stanczak-Mrozek et al., 2015), leads to a possible global adaptive benefit for certain clones and finally for the overall species, that is enhanced by HGT.

The necessary contribution of different genes for the full expression of AmpR phenotype is suggested by the lower MIC values of the transconjugants in comparison with the wild type strains in this and other studies (Rice et al., 2005b; Zhang et al., 2012). The transferable genetic platform identified here contained *pbp5* and *ponA* but also other genes (*ciaRH* operon) previously associated with β-lactam resistance and virulence in *S. pneumoniae*, but still unexplored in enterococci (Guenzi et al., 1994; Jordan et al., 2008; Krawczyk-Balska and Markiewicz, 2016). Outside this *pbp5*, only *ldt*_Efm_ and *ddcY* showed a similar non-congruent topology with that of *pbp5* indicating that these genes are also under accelerated evolution, which could explain eventual AmpR phenotypes with a lack of correlation with PBP5 sequences (Zhang et al., 2012). The variable ampicillin susceptibility phenotypes observed in different transconjugants even when using the same recipient strain, suggest either a partial transfer of the *pbp5* genetic platform or occurrence of recombination events leading to variation in MICs, which seem to occur frequently in commensal bacteria (Ingle et al., 2016).

## Conclusion

The arsenal of adaptive traits located on the chromosomal region containing *pbp5* characterized in this study suggests its involvement in the adaptation of *E. faecium* to the gastrointestinal tract of mammals, and its possible contribution to the spread of *pbp5* by horizontal gene transfer, even in the absence of specific β-lactam exposure. However, the apparent frequent transfer events of such genetic platform among “clouds” of closely related *E. faecium* populations, would indicate bacterial shifts in the evolution of pathogenicity (including colonization) and antibiotic resistance that occurred in response to changes in patients‘ demographics, medical strategies and interventions, within the paradigm of the “Hamiltonian medicine” (Fraser et al., 2005). The data further highlight the increasing need for evolutionary biology to be aligned with medical challenges (Nesse et al., 2010).

## Nucleotide Sequence Genbank Accession Numbers

The sequences corresponding to representative *pbp5* genetic plat forms (Types I-IV) were assigned GenBank accession numbers JN208885, JN208888, JN208884 and JN208886, respectively. PBP5 amino acid were analyzed and designated by a “C” followed by a number (Table S6). New sequences correspond to GenBank accession numbers JN208883 (C6 amino acid combination), JN208889 (C19 amino acid combination); JN208887 (C8 amino acid combination); JN208886 (C3 amino acid combination), JN208882 (C2 amino acid combination), KC479673 (C1 amino acid combination), KC479675 (C5 amino acid combination), KC479676 (C10 amino acid combination) and KC479674 (C12 amino acid combination). The Whole Genome Shotgun project has been deposited at DDBJ/ENA/GenBank under the accession MBRG00000000. The version described in this paper is version MBRG01000000.

## Author Contributions

CN, LP, and TMC designed the study. CN, ARF, APR, MA-H, ES, and RE performed wet lab experiments and participated in the analysis of the data. APT and VFL performed bioinformatic analysis. FB, APR, and LP provided expertise, participated in the analysis of data, and in the revision of the manuscript. CN, APT, and TMC performed the analysis of data and wrote the manuscript. All authors approved the final version of the manuscript.

## Conflict of Interest Statement

The authors declare that the research was conducted in the absence of any commercial or financial relationships that could be construed as a potential conflict of interest.

Authors have submitted the ICMJE Form for Disclosure of Potential Conflicts of Interest. Conflicts that the editor consider relevant to the content of the manuscript have been disclosed.

## Footnotes

1 http://pubmlst.org/

2 http://www.uniprot.org/uniref/

3 http://www.genome.jp/kegg/genome.html.

4 www.megasoftware.net
